# Environmental Factors Associated With Celiac Disease in Children in Southeast Iran: A Case–Control Study

**DOI:** 10.1155/ijpe/8824734

**Published:** 2025-10-24

**Authors:** Elahenaz Parsimood, Yeganeh Azhdary Moghaddam, Touran Shahraki, Ali Nemati

**Affiliations:** ^1^Department of Cardiology, School of Medicine, Kerman University of Medical Sciences, Kerman, Iran; ^2^Department of Pediatrics, School of Medicine, Zahedan University of Medical Sciences, Zahedan, Iran; ^3^Children and Adolescents Health Research Center, Research Institute of Cellular and Molecular Sciences in Infectious Diseases, Zahedan University of Medical Sciences, Zahedan, Iran; ^4^Department of Internal Medicine, School of Medicine, Kerman University of Medical Sciences, Kerman, Iran

**Keywords:** autoimmune disease, celiac disease, childhood, environmental factors, maternal factors, perinatal factor

## Abstract

**Background:**

Celiac disease (CD) is a systemic autoimmune disease associated with several environmental factors in susceptible individuals. Given the lack of CD-related publications in the Mediterranean region as well as its prevalence in southeastern Iran, we decided to assess its potential risk factors.

**Methods:**

In this case–control study, 200 children (100 CD and 100 non-CD patients) were recruited via convenience sampling. A survey assessing demographic and medical history (especially perinatal and maternal characteristics) was completed. Then, the odds ratios (ORs) were calculated through conditional logistic regression.

**Results:**

The risk of developing CD increased based on maternal BMI < 18.5 and > 30 kg/m^2^ (OR: 4.2, 95% CI: 1.3–13.2, and OR: 1.5, 95% CI: 0.6–3.7, respectively), living in rural areas (OR: 6.2, 95% CI: 2.6–14.2), use of antacids (OR: 7.4, 95% CI: 0.9–61.7), positive family histories of CD (OR: 11.8; 95% CI: 4.0–34.9), consanguine marriages (OR: 1.9, 95% CI: 1.1–3.5), and exclusive breastfeeding for 3 months (OR: 11, 95% CI: 1.3–87.6). Furthermore, premature rupture of membranes (OR: 0.489, 95% CI: 0.2–0.9) was associated with lower risks of CD.

**Conclusions:**

There is a need to increase awareness of the factors associated with CD in both society and among healthcare providers, to not only reduce exposure but diagnose quicker.

## 1. Introduction

Celiac disease (CD) is a systemic autoimmune disease characterized by the atrophy of the small intestinal mucosa upon exposure to gluten in genetically susceptible individuals [1]. In recent decades, the incidence of CD has increased significantly worldwide. Recent studies conducted in Iran indicate that the overall rate of CD (confirmed by biopsy) is 2%, which is higher than the reports from developed countries [2, 3]. Furthermore, improved diagnostic methods (such as serological testing, upper gastrointestinal [GI] endoscopy, and HLA availability), as well as increased overall awareness about the various clinical manifestations of CD, have accelerated patient detection [4, 5].

Although gluten ingestion and genetic susceptibility are required for the development of CD, some evidence suggests that environmental factors may also influence its onset [6, 7]. In addition, it has been proposed that infant diet (breastfeeding or formula feeding), mode of delivery, and maternal and perinatal conditions may be potential risk factors for CD [8–10]. However, these risk factors remain controversial [11–13]. Same with these factors, recent studies have shown that the use of antacids (such as proton pump inhibitors [PPIs] and histamine-2 receptor agonist) may contribute to the development of autoimmune and allergic conditions, including CD. Indeed, PPIs can lead to gut dysbiosis and enhance gluten immunopathology and inflammation [14–18].

The southeastern region of Iran has a high prevalence of CD patients [19]. However, no studies have investigated environmental, maternal, and perinatal factors in this region. Moreover, despite various studies on the mentioned factors, there is still no satisfactory explanation. Therefore, the purpose of our study is to evaluate some potential risk factors in our region to find the differences.

## 2. Methods

### 2.1. Research Design

This study used a case–control design to describe the relationship between maternal and perinatal environmental factors and CD. This study was conducted at the clinics of Ali-Asghar Children's Hospital, Zahedan University of Medical Sciences (ZAUMS), Zahedan, Iran.

### 2.2. Participants and Sampling

In this study, 200 children who were referred to Ali-Asghar Children's Hospital were selected from January 2019 to July 2019. This included 100 patients for the case group (with confirmation of CD by biopsy) and 100 healthy individuals for the control group (nonceliac patients with common cold complaints referring to the outpatient clinics). The population was selected using convenience sampling. Inclusion criteria were (1) age between 4 and 16 years, (2) a confirmed diagnosis of CD based on the European Society for Pediatric Gastroenterology Hepatology and Nutrition (ESPGHAN) guidelines (requiring villous atrophy on a normal diet, followed by clinical remission on a gluten-free diet), and (3) consent to participate and provide informed consent. Exclusion criteria were a history of metabolic, systemic, or other autoimmune diseases.

### 2.3. Data Collection

First, participants were given a consent form as well as multiple-choice questions about the consent process to ensure they understood the purpose and extent of the study. After providing the informed consent, participants were enrolled in the study to complete the questionnaire of environmental factor.

The questionnaire covered 24 characteristics selected based on the recommendations from previous studies as plausible factors of CD etiology and/or potential risk factors for immune system development during childhood. These factors include (1) maternal factors: maternal age, parental education level (that is divided into nonacademic individuals without any university education and academic ones who experience university education), premature rupture of membranes (PROM), smoking during pregnancy, maternal body mass index (BMI) (measured at first antenatal visit and divided into underweight [< 18.5 kg/m^2^], normal weight [18.5–24.9 kg/m^2^], overweight [25.0–29.9 kg/m^2^], and obesity [≥ 30.0 kg/m^2^]), and mode of delivery (vaginal, elective caesarean, and emergency caesarean); (2) perinatal factors: season of birth, duration of exclusive breastfeeding, age at first gluten introduction, number of family members, history of child antacid usage, familial marriage, region of residence, maternal infection during pregnancy, parity, and history of CD among other siblings; and (3) neonatal factors: gender, neonatal birth, small for gestational age, and neonatal infection.

### 2.4. Statistical Analysis

We used descriptive statistics to analyze the demographic characteristics. The distributions of quantitative data were assessed for normality (Kolmogorov–Smirnov test). Bivariate analysis using logistic regression (backward conditional) was performed to determine the independent association between CD and the explanatory variables. Multivariate logistic regression (backward conditional) analysis was performed for a number of explanatory variables and thereby reduced any potential bias due to differences between the compared groups. Multivariate logistic regression models included variables that were statistically significant in their prior bivariate models. For categorical variables, all categories were retained as long as there was a category that was statistically significant in the bivariate analysis. All analyses were repeated separately for boys and girls, except in the post hoc analysis. Odds ratios (ORs) with 95% confidence intervals (CIs) were calculated to estimate the risk of developing CD in the context of different conditions. CIs were calculated at the *p* value of 0.05, and analyses were performed using SPSS Statistics Version 26 (IBM Corporation, Chicago, Illinois, United States).

### 2.5. Ethical Considerations

This study adhered to the tenets of the Declaration of Helsinki and was ethically approved by Research Ethics Committees of ZAUMS (IR.ZAUMS.REC.1396.282). In addition, informed consents were obtained from the children and their parents.

## 3. Results

### 3.1. Demographic Findings and Factors Associated to CD

A total of 200 children (100 biopsy-proven CD patients as the case group and 100 children as the control group) were selected to evaluate. The case group included 40 males and 60 females (40% and 60%, respectively), while in the control group, the proportions were 51% and 49% (*p*: 0.118). Furthermore, the most common age range in the case and control groups was 58% under 7 years in the CD patient group and 51% between 7 and 12 years in the control group (Table 1).

Risk factors associated with the development of CD were exclusive breastfeeding (*p* = 0.007), PROM (*p* = 0.033), consumption history of antacid (*p* = 0.03), residency in village (*p* < 0.001), parental family relationship (*p* = 0.02), and having a family history of CD (*p* < 0.001). No significant associations were found with maternal education level, maternal age at delivery, season of birth, smoking during pregnancy, maternal infection during pregnancy, neonatal infections, mode of delivery, mother BMI before pregnancy, age of gluten introduction, duration of exclusive breastfeeding, parity, history of prematurity, gender, infant birth weight, and ethnicity (Tables 1 and 2).

### 3.2. Bivariate and Multivariate Results for All Participants

The results of bivariate analysis showed that the occurrence of CD was significantly associated with maternal BMI < 18.5 kg/m^2^ (compared to 18.5–24.99 kg/m^2^, *p* = 0.002), maternal BMI > 30 kg/m^2^ (compared to 25–30 kg/m^2^, *p* = 0.02), residency in rural areas (compared to city, *p* < 0.001), antacid usage (compared to lack of antacid, *p* = 0.03), positive family history of CD (compared to no family history, *p* < 0.001), parental family relationship (compared to no familial relationship, *p* = 0.02), exclusive breastfeeding just for 3 months (compared to exclusive breastfeeding for 2 years, *p* = 0.005) (Table 3). Additionally, reduced occurrence of CD was independently associated with PROM (compared to no PROM, *p* = 0.03) and exclusive breastfeeding (compared to no exclusive breastfeeding, *p* = 0.007). There was a trend toward an association between CD risk and parity; however, it was not significant.

No independent association was found between the occurrence of CD and maternal smoking, number of family members, parental education, maternal age less than 25 years, 25–29 years, and 30–35 years, maternal overweight or obesity, mode of delivery, age at gluten introduction, low birth weight, being small for gestational age, season of birth, neonatal infections, maternal infections, and ethnicity.

## 4. Discussion

The results of our study showed that the risk of CD during childhood is related to several environmental factors (perinatal and maternal). Perinatal characteristics associated with CD included the age younger than 7 years, living in a rural area (OR: 6.21, 95% CI: 1.97–15.15), consumption of antacid (OR: 7.45, 95% CI: 0.90–61.72), short-term breastfeeding duration, less than 3 months (OR: 11.0, 95% CI: 1.38–87.64), and history of CD in family members (OR: 11.82, 95% CI: 4–34.93). In addition, several maternal characteristics increased the risk of developing CD, which are as follows: maternal BMI less than 18.5 kg/m^2^ (OR: 4.23, 95% CI: 1.35–13.25) and greater than 30 kg/m^2^ (OR: 1.51, 95% CI: 0.615–3.713) and consanguine marriage (OR: 1.97, 95% CI: 1.10–3.51). However, exclusive breastfeeding (OR: 0.442, 95% CI: 0.242–0.806), residing in urban areas (OR: 0.161, 95% CI: 0.07–0.371), history of PROM (OR: 0.489, 95% CI: 0.252–0.948), and maternal age under 25 years old (OR: 0.06, 95% CI: 0.375–0.851) are related to lower CD occurrence.

Current research indicated that lower maternal age (especially under 25 years) may have a protective effect against CD. However, Sandberg et al. [20] and Namatovu et al. reported that older maternal age was associated with a lower occurrence of CD, and the authors attributed this correlation to infant feeding practices and aspects of lifestyle such as greater experience with exclusive breastfeeding and knowledge about the right time to introduce gluten. The reason for this difference may be due to increased attention and awareness among mothers in recent years as a result of patient education in southeastern Iran. Furthermore, our results showed that maternal BMI lower than 18.5 kg/m^2^ was associated with a higher incidence of CD in their children. According to the results of a Swedish study, maternal BMI in the normal and overweight ranges had a protective effect against the occurrence of CD, similar to our findings [10]. One limitation of our study was the lack of data about their nutritional status, past medical history, and GI symptoms. It is well established that underweight women are more likely to be malnourished or have abnormalities in their gut microbiota. Additionally, CD is also associated with other GI diseases like inflammatory bowel disease (IBD), fructose and lactose intolerance, and IgA deficiency [21–23]. Therefore, to determine the exact reason, we recommend further studies to identify maternal-related factors of CD patients.

Our results showed a significant relationship between factors related to infant feeding (exclusive milk feeding and breastfeeding duration) and CD. Radlović et al. (2010) stated that prolonged and continued breastfeeding after introducing gluten to the diet delays the onset of CD [24]. Breast milk, by providing protective immunological components (such as secretory IgA and cytokines) and by supporting the growth of beneficial microbiota (like *Bifidobacterium* spp. and *Lactobacillus* spp. that help oral tolerance to gluten), may enhance GI immune regulation and reduce the risk of immune dysregulation causing CD [25, 26]. Similar to our results, Szajewska et al. [27], Silano et al. [28], Virezinga et al. [29], and Lionetti et al. [30] stated that no existing evidence shows a reduced risk of CD with early or late gluten exposure in either the general population or in children at risk for CD. However, the current cohort studies have not determined a definite relationship between diet and CD occurrence.

Recent studies have emphasized more the association between breast milk, vaginal, and fecal microbiota, and the development of CD. Leonard et al. [31], Benitez-Paez et al. [8], and Štšepetova et al. [26] concluded that maternal breast milk microbiota can influence gut microbiota, immune markers, and the occurrence of CD. In addition, the mode of delivery showed no association with the CD onset. Though not significantly, emergency cesarean sections are shown to increase the risk of CD. Namatovu et al. [10] found that elective C-sections increase the risk of CD as infants born this way are not exposed to vaginal and fecal microbiota. However, two independent double-blind, placebo-controlled clinical trials found that vaginal delivery offered no protection against the onset of CD [29, 30]. Furthermore, a history of PROM (which is one of the main causes of exposure to vaginal microbiota) was associated with a reduced risk of CD. However, this finding was not consistent with the results of Namatovu et al. [10] and Koletzko et al. [9].

There was also a significant relationship between parental consanguinity and CD in siblings, both of which suggest a genetic basis for the CD. The risk of developing CD in first-degree relatives of CD is as high as 1.6%–38% [32], emphasizing the importance of family screening in reducing the underdiagnosis of CD. The importance of CD screening becomes clearer when we consider that nearly 30% of symptomatic first-degree relatives of CD patients in the United States remain undiagnosed [33]; therefore, due to the high prevalence of CD in the southeastern region of Iran, healthcare providers have to be familiar with the clinical manifestations and diagnostic methods of CD.

We found a significant relationship between living in rural areas and CD. Furthermore, the risk of CD was also higher in larger families (families of four to six members compared to families with three people) and higher numbers of parities. We may conclude that lower socioeconomic status (that often has more family members and living in countries) is associated with higher risks of CD, which could be due to reduced parental care and lower breastfeeding duration. Roy et al. [34] and Zingone et al. [35] concluded that children living in disadvantaged and underserved areas are more likely to be diagnosed with CD, as a result of less general population knowledge of CD, lower rates of health-seeking behaviors, and difficulty accessing health services.

The last factor associated with the occurrence of CD was the consumption of antacids. CD has been linked with several esophageal diseases like gastroesophageal reflux disease (GERD) and eosinophilic esophagitis (EoE) as a result of the GI epithelium's exposure to gluten [36]. Therefore, GI symptoms (leading the child to use antacid drugs) should be considered an important manifestation of CD.

## 5. Conclusion

In this study, we demonstrated that maternal BMI below 18.5 kg/m^2^, exclusive breastfeeding (for less than 3 months), consanguineous marriage, antacid use, residence in rural areas, family history, emergency cesarean section, and PROM increase the risk of CD. Therefore, raising families' awareness of preventable factors such as early gluten introduction, breastfeeding duration, antacid usage, and consanguineous marriage may reduce the incidence of CD in children. Furthermore, due to the large number of CD patients, healthcare providers need to be aware of clinical manifestations and diagnostic methods of CD for quicker identification.

## 6. Limitation and Strength of the Study

• The limitation of this study was a lack of access to some maternal information, which caused some CD patients to be excluded from the study.
• The strength of the study was the presence of biopsy-proven CD patients from the entire southeastern region of Iran. Ali-Asghar Children's Hospital, Zahedan, Iran, is the referral pediatric GI hospital in Sistan and Baluchestan Province, Iran. Therefore, the number of CD patients and the diversity of demographic characteristics provided optimal statistical power to ensure high precision in our estimates and allow adjustment for several variables.

## Figures and Tables

**Table 1:** Perinatal characteristics for case and control groups.

**Variables**	**Subgroups**	**Case group ** **N** **(%) (** **t** **o** **t** **a** **l** = 100**)**	**Control group ** **N** **(%) (** **t** **o** **t** **a** **l** = 100**)**	**p** **value**
Gender	Male	40 (40)	51 (51)	0.118
	Female	60 (60)	49 (49)	

Infant birth weight (grams)	< 1500	5 (5)	2 (2)	0.383
	1500–2499	33 (33)	29 (29)	
	≥ 2500	62 (62)	69 (69)	

Preterm	Yes	10 (10)	6 (6)	0.297
	No	90 (90)	94 (94)	

Season of birth	Spring	24 (24)	24 (24)	0.971
	Summer	36 (36)	33 (33)	
	Autumn	14 (14)	15 (15)	
	Winter	26 (26)	28 (28)	

Kid age (year)	< 7	58 (58)	38 (38)	0.018
	7–12	34 (34)	51 (51)	
	> 13	8 (8)	11 (11)	

Duration of breastfeeding	First 3 months	10 (10)	1 (1)	0.48
	First 6 months	8 (8)	9 (9)	
	First year	27 (27)	32 (32)	
	Two years	55 (55)	58 (58)	

Exclusive breastfeeding	Yes	57 (57)	75 (75)	0.007
	No	43 (43)	25 (25)	

Age at gluten introduction (month)	< 5	14 (14)	8 (8)	0.221
	5–7	76 (76)	76 (76)	
	> 8	10 (10)	16 (16)	

Residency	Urban area	65 (65)	92 (92)	0.001
	Rural area	35 (35)	8 (8)	

Parity	1	27 (27)	40 (40)	0.243
	2	28 (28)	26 (26)	
	3	20 (20)	16 (16)	
	≥ 4	25 (25)	18 (18)	

Family members	< 4	23 (23)	33 (33)	0.251
	4–6	52 (52)	48 (48)	
	> 7	25 (25)	19 (19)	

History of antacid use	Yes	7 (7)	1 (1)	0.03
	No	93 (93)	99 (99)	

Parental family relationship	Yes	69 (69)	53 (53)	0.02
	No	31 (31)	47 (47)	

Ethnicity	Baloch	66 (66)	61 (61)	0.503
	Sistani	28 (28)	35 (35)	
	Others	6 (6)	4 (4)	

**Table 2:** Maternal characteristics for case and control groups.

**Variables**	**Subgroups**	**Case group** **N** **(%) (** **t** **o** **t** **a** **l** = 100**)**	**Control group** **N** **(%) (** **t** **o** **t** **a** **l** = 100**)**	**p** **value**
Maternal age	< 25	54 (54)	64 (64)	0.340
	25–29	17 (17)	16 (16)	
	30–35	15 (15)	13 (13)	
	> 35	14 (14)	7 (7)	

Mother's BMI	< 18.5	15 (15)	4 (4)	0.023
	18.5–24.99	51 (51)	55 (55)	
	25–30	21 (21)	32 (32)	
	> 30	13 (13)	9 (9)	

Father's education	Nonacademic	89 (89)	84 (84)	0.301
	Academic	11 (11)	16 (16)	

Mother's education	Nonacademic	92 (92)	86 (86)	0.175
	Academic	8 (8)	14 (14)	

Mode of delivery	Vaginal	72 (72)	75 (75)	0.306
	Elective caesarean	9 (9)	13 (13)	
	Emergency caesarean	19 (19)	12 (12)	

PROM	Yes	18 (18)	31 (31)	0.033
	No	82 (82)	69 (69)	

Smoking during pregnancy	Yes	5 (5)	3 (3)	0.450
	No	95 (95)	97 (97)	

Maternal infections	Yes	32 (32)	31 (31)	0.879
	No	68 (68)	69 (69)	

Neonatal infections	Yes	18 (18)	20 (20)	0.718
	No	82 (82)	80 (80)	

**Table 3:** Celiac disease relation to maternal and perinatal characteristics (bivariate and multivariate analysis).

**Variables**	**Subgroups**	**Bivariate**		**Multivariate**	
		**OR (95% CI)**	**p** **value**	**OR (95% CI)**	**p** **value**
Gender	Male	0.641 (0.366–1.122)	0.118	0.948 (0.374–2.406)	0.911
	Female				

Season	Spring	1 (0.523–1.914)	1	1	0.462
	Summer	1.142 (0.637–2.047)	0.655	1.450 (0.539–3.904)	0.793
	Autumn	0.922 (0.420–2.028)	0.841	1.170 (0.361–3.796)	0.559
	Winter	0.903 (0.484–1.687)	0.750	0.683 (0.189–2.459)	

Premature	Yes	1.741 (0.608–4.987)	0.297	3.324 (0.856–12.91)	0.083
	No				

Infant birth weight (grams)	< 1500	2.579 (0.488–13.617)	0.248	1	0.221
	1500–2499	1.206 (0.662–2.198)	0.541	3.868 (0.443–33.74)	0.849
	≥ 2500	0.733 (0.408–1.316)	0.298	1.101 (0.409–2.961)	

Duration of breastfeeding (month)	First 3 months	11 (1.381–87.641)	0.005	1	0.048
	First 6 months	0.879 (0.325–2.379)	0.800	12.012 (1.027–140.473)	0.065
	First year	0.786 (0.427–1.446)	0.438	0.231 (0.049–1.097)	0.603
	Two years	0.885 (0.506–1.548)	0.669	0.767 (0.283–2.080)	

Age at gluten introduction (month)	< 5	1.872 (0.748–4.684)	0.175	1	0.513
	5–7	1 (0.523–1.914)	1	1.633 (0.375–7.107)	0.483
	> 8	0.583 (0.251–1.357)	0.207	1.578 (0.442–5.63)	

Parity	1	0.555 (0.306–1.007)	0.051	1	0.626
	2	1.107 (0.593–2.067)	0.75	1.594 (0.244–10.401)	0.256
	3	1.312 (0.636–2.710)	0462	1.729 (0.673–4.442)	0.547
	≥ 4	1.519 (0.768–3.003)	0.228	1.489 (0.408–5.439)	

Mother's age	< 25	0.66 (0.375–0.851)	0.151	1	0.009
	25–29	1.075 (0.509–2.270)	0.849	0.346 (0.156–0.768)	0.522
	30–35	1.181 (0.530–2.630)	0.684	0.645 (0.169–2.467)	0.425
	> 35	2.163 (0.834–5.612)	0.106	0.492 (0.086–2.811)	

Mother's BMI	< 18.5	4.235 (1.353–13.25)	0.008	1	0.002
	18.5–24.99	0.852 (0.488–1.485)	0.571	9.741 (2.383–39.812)	0.172
	25–30	0.565 (0.298–1.070)	0.078	0.428 (0.127–1.445)	0.021
	> 30	1.511 (0.615–3.713)	0.036	0.572 (0.237–1.381)	

Number of family members	< 4	0.606 (0.325–1.133)	0.115	1	0.002
	4–6	1.174 (0.674–2.044)	0.572	0.545 (0.196–1.514)	0.172
	> 7	1.421 (0.724–2.789)	0.306	0.684 (0.224–2.085)	0.021

Exclusive breastfeeding	Yes	0.442 (0.242–0.806)	0.007	0.24 (0.099–0.584)	0.002
	No				

Mother's education	Yes	1.872 (0.748–4.684)	0.175	5.034 (1.325–19.129)	0.018
	No				

Father's education	Yes	1.541 (0.677–3.512)	0.253	1.505 (0.269–8.412)	0.642
	No				

Residency	Urban area	0.161 (0.070–0.371)	< 0.001	0.183 (0.066–0.506)	< 0.001
	Rural area				

History of antacid use	Yes	7.452 (0.900–61.72)	0.03	41.234 (2.943–577.668)	0.006
	No				

PROM	Yes	0.489 (0.252–0.948)	0.033	0.237 (0.090–0.628)	0.004
	No				

Celiac history in the family	Yes	11.821 (4.00–34.93)	< 0.001	12.781 (3.762–43.418)	< 0.001
	No				

Smoking during pregnancy	Yes	1 (0.280–3.567)	1	0.714 (0.082–6.208)	0.760
	No				

Mode of delivery	Vaginal	0.857 (0.457–1.607)	0.631	1	0.436
	Elective caesarean	0.662 (0.269–1.627)	0.366	3.200 (0.172-59-713)	0.481
	Emergency caesarean	1.720 (0.786–3.764)	0.171	0.721 (0.231–1.985)	

Maternal infections	Yes	1.047 (0.577–1.902)	0.879	0.698 (0.255–1.910)	0.484
	No				

Parental family relationship	Yes	1.974 (1.108–3.517)	0.02	1.359 (0.537–3.441)	0.517
	No				

Ethnicity	Baloch	1.241 (0.697–2.209)	0.463	1	0.261
	Sistani	0.755 (0.414–1.378)	0.359	1.723 (0.667–4.450)	0.703
	Others	2.064 (0.502–8.493)	0.306	1.459 (0.209–10.166)	

## Data Availability

The datasets used and/or analyzed during the current study are available from the corresponding author on reasonable request.
